# NLRP1 inflammasome is activated in patients with medial temporal lobe epilepsy and contributes to neuronal pyroptosis in amygdala kindling-induced rat model

**DOI:** 10.1186/s12974-014-0233-0

**Published:** 2015-01-28

**Authors:** Chen-Chen Tan, Jian-Guo Zhang, Meng-Shan Tan, Hua Chen, Da-Wei Meng, Teng Jiang, Xiang-Fei Meng, Ying Li, Zhen Sun, Meng-Meng Li, Jin-Tai Yu, Lan Tan

**Affiliations:** Department of Neurology, Qingdao Municipal Hospital, School of Medicine, Qingdao University, No.5 Donghai Middle Road, Qingdao, 266071 China; Department of Functional Neurosurgery, Beijing Neurosurgical Institute, Capital Medical University, No.6, Tiantan Xili, Beijing, 100050 China; Department of Neurosurgery, Beijing Tiantan Hospital, Capital Medical University, No.6, Tiantan Xili, Beijing, 100050 China; College of Medicine and Pharmaceutics, Ocean University of China, No.5 Yushan Road, Qingdao, 266003 China; Department of Pathology, Qingdao Municipal Hospital, School of Medicine, Qingdao University, No.5 Donghai Middle Road, Qingdao, 266071 China; Department of Neurology, Qingdao Municipal Hospital, Nanjing Medical University, No.5 Donghai Middle Road, Qingdao, 266071 China; Memory and Aging Center, Department of Neurology, University of California, San Francisco, CA 94158 USA

**Keywords:** NLRP1, pyroptosis, inflammasome, Caspase-1, temporal lobe epilepsy

## Abstract

**Background:**

Temporal lobe epilepsy (TLE) is often characterized pathologically by severe neuronal loss in the hippocampus. Understanding the mechanisms of neuron death is key to preventing the neurodegeneration associated with TLE. However, the involvement of neuronal loss to the epileptogenic process has yet to be fully determined. Recent studies have shown that the activation of NLRP1 can generate a functional caspase-1-containing inflammasome *in vivo* to drive the proinflammatory programmed cell death termed ‘pyroptosis’, which has a key role in the pathogenesis of neurological disorders. To the best of our knowledge, there are no reported studies that performed detailed identification and validation of NLRP1 inflammasome during the epileptogenic process.

**Methods:**

We first compared expression of NLRP1 and caspase-1 in resected hippocampus from patients with intractable mesial temporal lobe epilepsy (mTLE) with that of matched control samples. To further examine whether the activation of NLRP1 inflammasome contributes to neuronal pyroptosis, we employed a nonviral strategy to knock down the expression of NLRP1 and caspase-1 in the amygdala kindling-induced rat model. Proinflammatory cytokines levels and hippocampal neuronal loss were evaluated after 6 weeks of treatment in these NLRP1 or caspase-1 deficiency TLE rats.

**Results:**

Western blotting detected upregulated NLRP1 levels and active caspase-1 in mTLE patients in comparison to those levels seen in the controls, suggesting a role for this inflammasome in mTLE. Moreover, we employed direct *in vivo* infusion of nonviral small interfering RNA to knockdown NLRP1 or caspase-1 in the amygdala kindling-induced rat model, and discovered that these NLRP1 or caspase-1 silencing rats resulted in significantly reduced neuronal pyroptosis.

**Conclusions:**

Our data suggest that NLRP1/caspase-1 signaling participates in the seizure-induced degenerative process in humans and in the animal model of TLE and points to the silencing of NLRP1 inflammasome as a promising strategy for TLE therapy.

**Electronic supplementary material:**

The online version of this article (doi:10.1186/s12974-014-0233-0) contains supplementary material, which is available to authorized users.

## Background

Temporal lobe epilepsy (TLE), a serious, chronic neurological syndrome in patients presenting refractory seizures, is characterized pathologically by selective neuronal cell loss and mossy fiber sprouting in hippocampus and limbic system. Hippocampal sclerosis (HS) is the most common lesion found in patients with mTLE. Experimental studies in animal models of epilepsy and human brain tissue have revealed a role of progressive damage and considerable neuronal death in vulnerable areas such as the hippocampus of TLE [1,2]. However, the exact molecular pathogenic mechanisms of neuronal loss are not completely understood.

Recently, a novel inflammasome signaling pathway has been uncovered, and a wealth of information linking the activation of inflammasome to neurological disorders pathogenesis has emerged [3]. The NLRP1 inflammasome was first characterized as a member of the NLRP family, whose activation can generate a functional caspase-1-containing inflammasome *in vivo* to drive the proinflammatory programmed cell death termed ‘pyroptotic death’ [4]. Like apoptosis, pyroptosis requires the proteolytic activation of specific caspases: caspase 3 and 7 for apoptosis and caspase-1 for pyroptosis. In contrast to apoptosis, which is often anti-inflammatory, pyroptosis is predicted to be proinflammatory due to the rapid loss of cell membrane integrity and release of cytosolic contents [5]. In addition, inhibition of the NLRP1 inflammasome could reduce the innate immune response and ameliorate detrimental consequences inflammation [6,7]. As a critical component of the inflammasome, NLRP1 appears to be expressed rather ubiquitously, and high NLRP1 levels were also found in the brain, in particular in pyramidal neurons and oligodendrocytes [8]. Although current data regarding NLRP1 functions are far scarcer than those that described for other inflammasome, various studies have proposed its crucial role in neurological diseases such as neurodegenerative pathologies, in which inflammatory events and neuronal death have a clear causal role [9]. Thus, we hypothesized that the activation of NLRP1-inflammasome may have key roles in the pathogenesis of TLE, in which inflammatory events and neuronal death contribute powerful pathogenetic forces [10,11]. To test this hypothesis, we first investigated whether the expression profiles of the NLRP1 inflammasome components, including NLRP1 and caspase-1, are altered in resected hippocampus from patients with intractable mTLE when compared to matched control samples. Next, we applied small interfering RNAs (siRNAs) to knock down NLRP1 and caspase-1 in the brain of amygdala kindling-induced TLE rat model and measured the NLRP1 component alterations as well as the functional outcomes.

## Methods

### Temporal lobe epilepsy patients and control group

We collected the hippocampus samples of mTLE patients. Patients recruited in this study have a diagnosis of refractory epilepsy according to the definition of pharmacoresistant epilepsy [12]. The 24-h EEG monitoring indicated that widespread sharp or spike or slow-waves originated from unilateral temporal lobe. In addition, there was ipsilateral hippocampal sclerosis identified by MRI or low metabolism in ipsilateral mesial temporal lobe identified by 18F-FDG PET, without other pathological changes. Surgery was determined by a neurologist and a neurosurgery specialist in consultation. During the operation, cortical electrode monitoring confirmed that epilepsy-like waves originated from the inferior temporal lobe. All patients showed HS, with appreciable neuronal loss and reactive gliosis in CA1, CA3 and CA4. It was infeasible to obtain brain tissues of normal hippocampus cortex, so that normal temporal cortex tissues were used as negative control. Considering the difficulty in finding ‘real’ controls in human studies, in our experiments we used ‘healthy’ surgical samples from patients with other pathologies. Seizure absence was determined by the patient’s report to the neurologist during the scheduled visits. Therefore, for comparative purposes, we used six specimens of nonepileptic tissues from Histologically normal specimens (control samples) in accordance with all legal requirements. None of the patients in the control group had a history of systematic diseases. We also used autopsy hippocampal tissue as a second control tissue for these studies to avoid problems due to the localization of specific expression. Autopsy hippocampal tissues from six patients with no known history of epilepsy and other systematic diseases were used as controls. The average postmortem interval was within 12 h after death. Routine neuropathological examination of tissue sections of these control hippocampal samples showed no pathologic changes. The brain tissues were separated as needed and immediately preserved in liquid nitrogen. Clinicopathological data are presented in Table 1, Additional file 1: Figure S1, and Additional file 2: Figure S2. The study was approved by the Ethics Committee of Qingdao Municipal Hospital. Informed consent was obtained from the patients and their legal guardians on the use of their brain tissues in this research.

**Table 1:** **Clinical data of temporal lobe epilepsy patient group and control groups**

**(a) Clinical data of temporal lobe epilepsy patient group**							
**Case**	**Sex**	**Age, y**	**Seizure type**	**Duration, y**	**EEG, sp ori**	**MRI/PET**	**HS**
1	M	27	CPS, SGS	24	R-T	R-HS/R-T^a^	Wyler III
2	F	24	CPS	22	L-T	L-HS/L-T^a^	Wyler IV
3	F	23	CPS	10	L-T	L-HS/ -	Wyler III
4	F	33	CPS	26	L-T	L-HS/ -	Wyler III
5	M	26	CPS	20	R-T	R-HS/R-T^a^	Wyler IV
6	F	26	CPS	10	L-T	L-HDA/L-T^a^	Wyler III
**(b) Clinical data of normal temporal cortex tissues control group**							
**Case**	**Sex**	**Age, y**	**Tissue sources**				
1	M	29	Operative route of benign neoplasm in deep area of brain				
2	M	38	Adjacent normal cortex in surgical evacuation of meningioma				
3	M	23	Operative route of benign neoplasm in deep area of brain				
4	F	37	Adjacent normal cortex in surgical evacuation of BGH				
5	F	31	Adjacent normal cortex in surgical evacuation of IH				
6	M	33	Operative route of benign neoplasm in deep area of brain				
**(c) Clinical data of postmortem hippocampal tissue control group**							
**Case**	**Sex**	**Age, y**	**Cause of death**				
1	M	45	Sudden cardiac death				
2	M	24	Car accident				
3	F	19	Car accident				
4	M	32	Pulmonary artery embolism				
5	F	25	Amniotic fluid embolism				
6	F	23	Amniotic fluid embolism				

BGH, basal ganglia hemorrhage; CPS, complex partial seizure; F, female; HAD, hippocampal degenerative atrophy; HS, hippocampal sclerosis, IH, intracerebral hematoma; L, left; M, male; R, right; SGS, secondarily generalized seizure; sp ori, spikes origin; T, temporal lobe; y, year.

^a^Indicates that the positron emission tomography (PET) shows low metabolism.

### Animals

To avoid the interference of estrogen on neuroinflammation [13], only male rats were used in this study. Adult male Sprague-Dawley rats weighing 260 to 300 g were obtained from the Experimental Animal Center of Qingdao University. They were housed in a standard animal room on a 12 h light/dark cycle with a controlled temperature and humidity, and given free access to food and water. All experiments were performed in strict accordance with the National Institute of Health Guide for the Care and Use of Laboratory Animals. Animal care and sacrifice were conducted according to methods approved by the Qingdao University Animal Experimentation Committee. All efforts were made to minimize the number of animals used and their suffering.

### Electrode implantation and temporal lobe epilepsy induction

Rats were positioned in a stereotaxic apparatus (Stoelting, USA, www.stoeltingco.com) under deep anesthesia (10% chloral hydrate, 3.5 mL/kg, i.p.). An electrode was implanted into the right basolateral amygdala (AP: -3.0 mm; L: -4.8 mm; V: -8.8 mm) [14,15]. The electrode was connected to a miniature receptacle, which were fixed to the skull using dental cement anchored with stainless steel screws. All rats underwent an identical surgical procedure, which were performed with the use of antiseptic technique.

After electrode implantation, the rats were allowed to recover for 2 weeks. Seizures were induced by a 20-minute amygdala stimulation (100 ms train of 1 ms, 60 Hz bipolar pulses, 400 μA, every 0.5 s) using a ML1101 electronic stimulator. Electroencephalograms of the right amygdala were recorded with a digital amplifier (AD Instrument, Racine, WI, USA). Following electrical stimulation, rats were video-monitored for 8 weeks. The rats with chronic TLE were identified by occurrence of frequent seizures (at least 2 times IV/V spontaneous seizures per week) from 1 week after electrical stimulation. Control rats were handled in the same manner but did not receive any electrical stimulation. Moreover, our preliminary experiments showed that this amygdala stimulation model is effective.

### siRNA administration in rat brain

We prepared the Entranster *in vivo*- siRNA mixture according to the manufacturer’s instructions. Briefly, 50 μg NLRP1 siRNA (Santa Cruz Biotechnology, Inc., Dallas, Texas, USA) or caspase-1 siRNA (Santa Cruz Biotechnology, Inc., Dallas, Texas, USA) or control siRNA (Santa Cruz Biotechnology, Inc., Dallas, Texas, USA) was resuspended in 50 μL RNAse-free water to make a siRNA solution. Then, 50 μL NLRP1 siRNA or control siRNA solution was mixed with 50 μL Entranster *in vivo* transfection reagent (Engreen, Inc., Beijing, China) and 100 μL artificial cerebrospinal fluid (aCSF, composition in mmol/L: NaCl 130, KCl 2.99, CaCl_2_ 0.98, MgCl_2_•6H_2_O 0.80, NaHCO_3_ 25, Na_2_HPO_4_•12H_2_O 0.039, NaH_2_PO_4_•2H_2_O 0.46) to get a 200-μL *in vivo* transfection mixture. NLRP1 siRNA or caspase-1 siRNA at this dose was well tolerated, and no signs of neurotoxicity including hind-limb paralysis, vocalization, food intake, or neuroanatomic damage were observed in the preliminary study.

We filled an osmotic pump (Model 2006; ALZET Inc., Cupertino, CA, USA) with the *in vivo* transfection mixture or aCSF alone. Meanwhile, rats were anesthetized with 10% chloral hydrate (0.3 mL/100 g, intraperitoneal) and were fixed in a stereotactic frame (Stoelting, USA, www.stoeltingco.com). A brain-infusion cannula (ALZET Inc., Cupertino, CA, USA) coupled via vinyl tubing to the osmotic pump was implanted into the dorsal third ventricle (AP: -1.8 mm; L: -0 mm; V: -5 mm) of rats with chronic TLE 8 weeks after electrical stimulation. Meanwhile, the osmotic pump was placed subcutaneously between the scapulae of rat, and the siRNAs were continuously infused into the brain at a flow rate of 0.15 μL/hour for 6 weeks by the osmotic pumps. All surgical procedures were ere performed with the use of antiseptic technique.

### Behavioral assessment of seizure

All animals were assessed for amygdala stimulation induced seizures during the first 24 h postsurgery. At the 8th week postsurgery, all animals were re-evaluated for behavioral progression of seizures 4 h/day for 5 consecutive days to record the spontaneous seizures and scored according to Racine’s classification [16]: 0, no reaction; 1, stereotypic mounting, eye blinking, and/or mild facial clonus; 2, head nodding and/or multiple facial clonus; 3, myoclonic jerks in the forelimbs; 4, clonic convulsions in the forelimbs with rearing; and 5, generalized clonic convulsions and loss of balance. Numbers and rates of animals with seizures (at least 2 times IV/V spontaneous seizures) during the first 24 h post-surgery and on the 6th week post-treatment were recorded.

### Brain tissue preparation

Rats were sacrificed under deep anesthesia for the following biochemical assays:

1. For western blot analysis, quantitative real-time polymerase chain reaction (PCR), and enzyme-linked immunosorbent assay (ELISA), the rats were perfused transcardially with 0.9% saline (pH7.4). Then, the samples were rapidly isolated and placed in liquid nitrogen until use.
2. For cresyl violet staining and TUNEL analysis, rats were perfused transcardially with 0.9% saline (pH 7.4), followed by a fixative solution containing 4% paraformaldehyde in PBS (pH 7.4). Then, the brains were removed and fixed in the same fixative at 4°C until use.
3. For double immunofluorescence staining, rat brain was removed without perfusion, embedded in tissue freezing medium, and immediately frozen at -40°C. Frozen tissue was stored at -80°C until sectioning.

### Western blotting

Tissues samples were digested with RIPA lysis buffer (50 mmol/L Tris-HCl, 150 mmol/L NaCl, 1% Nonidet-40, 0.5% sodium deoxycholate, 1 mmol/L EDTA, 1 mmol/L PMSF) with protease inhibitors (pepstatin 1 μg/mL, aprotinin 1 μg/mL, leupeptin 1 μg/mL) for 30 min and the protein concentration was determined using the Bradford assay kit (Bio-Rad Laboratories, Hercules, CA, USA). Different samples with an equal amount of protein were separated using 8-12% SDS-polyacrylamide gels and transferred to PVDF membranes. After blocking with 10% non-fat milk at room temperature, the membranes were incubated with primary antibodies against NALP1 (1:1000; Novus Biologicals, Inc., Littleton, CO, USA), NeuN (1:500; Chemicon, www.chemicon.com.br), cleaved caspase-1 (1:200; Cambridge, UK), cleaved IL-1β (1:500; Santa Cruz Biotechnology, Inc., Dallas, Texas, USA), and β-actin (1:1000; Santa Cruz Biotechnology, Inc., Dallas, Texas, USA) at 4°C overnight. After rinsing, the membranes were appropriately incubated with horseradish peroxidase (HRP)-conjugated suitable secondary antibodies (1:5000; Zhongshan Inc., Beijing, China) for 2 h at room temperature. Cross-reactivity was visualized using ECL western blotting detection reagents and analyzed by scanning densitometry using a BioSpectrum Imaging System (UVP, Upland, CA, USA).

### Real-time PCR

Total RNA was extracted using TRIzol reagent (Invitrogen Life Technologies, Carlsbad, CA, USA), using the protocol supplied by the manufacturer. Total RNA was reverse transcribed to cDNA by the Reverse Transcription System (Bio-Rad, Hercules, CA, USA). The reaction was performed at 42°C for 50 min, 95°C for 5 min, and 5°C for 5 min, then the cDNA was stored in -20°C. Amplification was carried out with the Stratagene Mx3000P real-time PCR system (Stratagene, LaJolla, CA, USA) with a SYBR Green PCR technology (Takara Bio, Inc., Shiga, Japan). Reverse transcription was performed in a final volume of 20 μL containing 2 μL cDNA, 10 μL SYBR Green, 0.4 μL ROX Reference Dye, 0.4 μL forward and reverse primer (1 mol/L), and 6.8 μL nuclease-free water. The optimal conditions were 40 cycles of 95°C for 30 s, 60°C for 32 s, and 72°C for 30 s. Relative quantification was given by the CT values, determined for triplicate reactions of SE samples and sham samples for each gene. Total RNA concentrations from each sample were normalized by quantity of β-actin mRNA, and the target genes’ expression was evaluated by ratio of the number of target mRNA to β-actin mRNA. Relative expression of genes was obtained by the 2^-△△CT^ method. Primers were purchased from Invitrogen as follows (name: forward primer, reverse primer): *nlrp1*: 5’-gccctggagacaaagaatcc-3’, 5’-agtgggcatcgtcatgtgt-3’; *caspase-1*: 5’-aaggtcctgagggcaaagag-3’, 5’-gtgttgcagataatgagggc-3’; *β-actin*: 5’-agggaaatcgtgcgtgac-3’, 5’-cgctcattgccgatagtg-3’.

### ELISA

Levels of secreted IL-1β were measured using commercially available ELISA kit (R&D Systems, Minneapolis, MN, USA) according to the manufacturer’s instructions. ELISA was conducted by technicians who were blinded to the experimental groups. The results are expressed in pg/mL.

### Nissl staining and TUNEL assay

Nissl staining was employed to detect surviving neurons. The brains were embedded in paraffin and cut into 7-μm sections. Next, the paraffin-embedded sections were dewaxed and rehydrated according to the standard protocols. Then, the sections were stained in 1% cresyl violet at 50°C for 5 minutes. After being rinsed with water, the sections were dehydrated in increasing concentrations of ethanol, mounted on the slides, and examined with a light microscope. Only the neurons with violet nucleus and the intact morphology were counted as surviving neurons. Besides, we used a cell death detection kit (In Situ Cell Death Detection Kit, POD; Roche, www.roche.com) for TUNEL assay to detect neuronal apoptosis. We employed TUNEL assay via a commercial kit according to the manufacturer instructions. Briefly, the paraffin-embedded sections of samples received deparaffinization and rehydration treatments, and then were incubated with proteinase-K for 15 min at room temperature followed by three washes in PBS. The TUNEL reaction mixture was added and incubated for 60 min at 37°C. Next, sections were washed with PBS and two drops of peroxidase-streptavidin conjugate solution in blocking buffer was applied. Then, the sections were incubated for 30 min at room temperature, washed again with PBS and exposed to 0.03% diaminobenzidine in 0.01% H_2_O_2_. Lastly, sections were counterstained with Mayer’s hematoxylin, dehydrated, mounted on the slides and examined with a microscope equipped with a charge-coupled device camera. We identified the neurons with deep black nuclei as TUNEL-positive neurons. Cell counting was performed on five randomly selected non-overlapping fields of per slide. The densities of surviving neurons or TUNEL-positive neurons in the hippocampus of the scanned digital images were calculated using Image-Pro Express software (Media Cybernetics, Silver Spring, MD, USA). The total cell counts were averaged from five sections per animal. The survival index was defined as follows: surviving index (%) = 100× (number of surviving neurons/total number of neurons). Furthermore, the TUNEL-positive neurons index was defined as follows: TUNEL-positive neurons index (%) = 100× (TUNEL-positive neurons/total neurons).

### Immunofluorescence staining

For immunofluorescence studies, 20-μm-thick sections were obtained by cryosectioning at -20°C, mounted on a glass slide, and incubated at room temperature for 1 hour. Next, the sections were fixed in ice-cold acetone for 10 minutes and then dried on a heater for 10 minutes at 40°C. Then, sections were blocked with 5% BSA and 0.1% TritonX-100 for 2 hours. After a single wash with PBS, sections were incubated overnight at 4°C with a rabbit polyclonal antibody against NALP1 (1:1000; Novus Biologicals, Inc., Littleton, CO, USA) combined with a mouse monoclonal antibody against NeuN (1:200; Chemicon, www.chemicon.com.br). Afterward, sections were washed in PBS and incubated respectively with FITC conjugated anti-rabbit IgG (1:200; Santa Cruz Biotechnology, Inc., Dallas, Texas, USA) and TRITC conjugated anti-mouse IgG (1:200; Zhongshan Inc., Beijing, China) in a dark and humidified container for 1 h at 37°C. After that, the sections were washed with PBS and sealed with a cover slip. The slides were analyzed with a fluorescence microscopy (Olympus, Inc, Tokyo, Japan). To ensure the specificity of the immunoblotting procedure, control experiments were performed in which the corresponding primary antibody was omitted. Under these conditions, no signal was observed.

### Statistical analysis

Statistical analysis was conducted by SPSS software 17.0 (IBM, Inc., Armonk, NY, USA). After confirming normal distribution with the skewness and kurtosis statistic test, an independent sample t-test or one-way ANOVA followed by a least significant difference (LSD) post hoc test was used to analyze differences among groups. All data are expressed as mean ± standard deviation. *P* <0.05 was considered statistically significant.

## Results

### NLRP1 inflammasome components were upregulated in human mesial temporal lobe epilepsy

**Figure 1:**
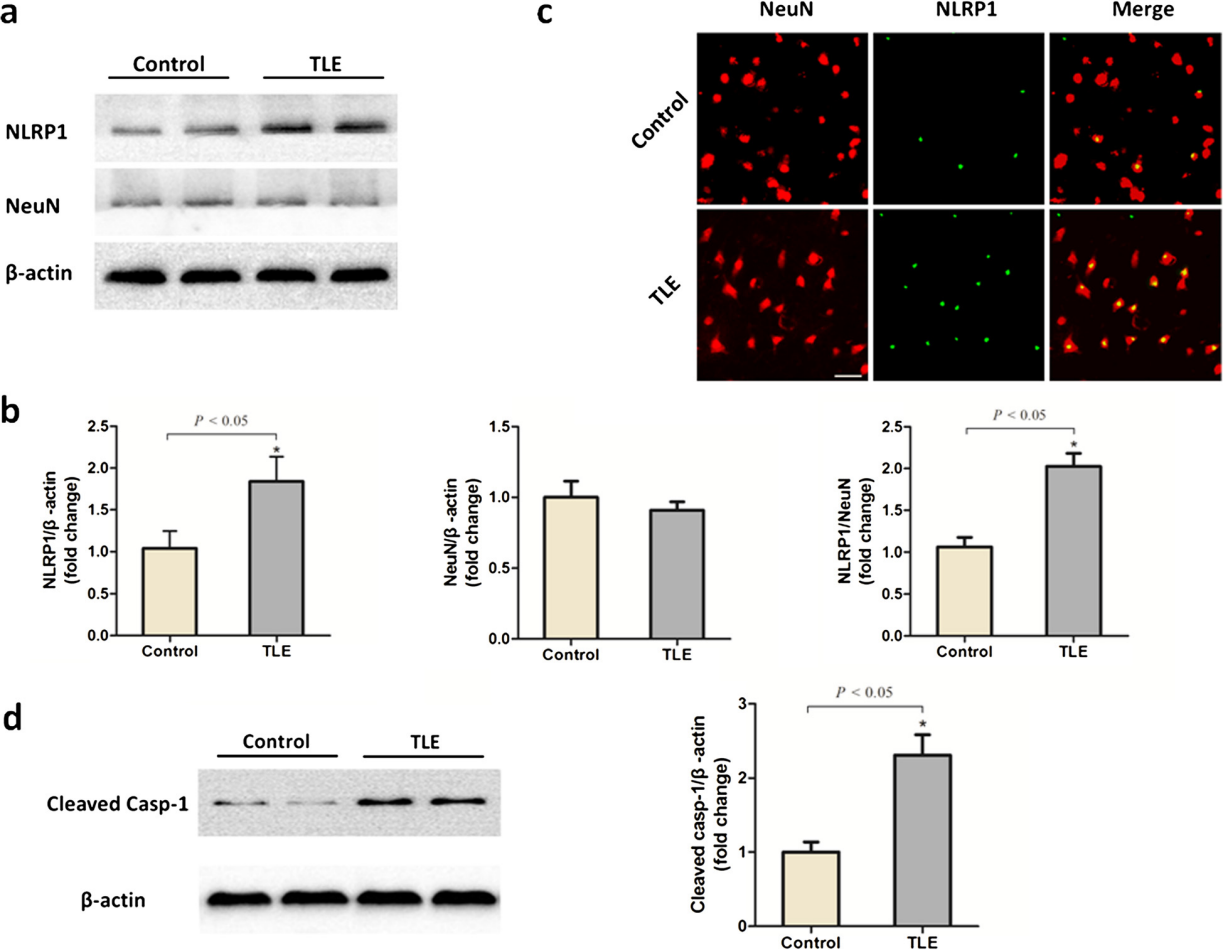
**Increased expression of NLRP1 and caspase-1 in the neurons of temporal lobe epilepsy (TLE) patient brains. (a)** Cerebral NLRP1 and NeuN levels from Case 1 TLE patient and Case 1 ‘healthy control’ individual were detected by western blot analysis. β -actin was used as loading control. **(b)** Levels of NLRP1, NeuN and NLRP1/NeuN were quantified by densitometric measurement. Values are the mean ± standard deviation. **(c)** Double immunofluorescent detection of NLRP1 in the NeuN-positive neurons of Case 6 TLE patient and Case 6 ‘healthy control’ individual. **(d)** The expression level of active caspase-1 (20KD) in Case 3 TLE patient and Case 3 ‘healthy control’ individual was analyzed using the western blot assay. Tissue samples from hippocampus of the TLE group and control group were immunostained using anti-NLRP1 and anti-NeuN antibodies and examined under a fluorescence microscope. Scale bars: 20 μm. n = 6 individuals per group.

We first investigated whether the expression of NLRP1 was altered in the brains of individuals with pharmacoresistant mTLE. Total proteins were extracted from the surgically obtained hippocampus samples and control samples and subjected to western blot analysis. We found that the NLRP1 levels of TLE patients were significantly elevated, while the levels of NeuN were slightly reduced compared to control samples (Figure 1a, b). Using double immunofluorescence staining to colocalize NLRP1 with neuronal marker NeuN, our results further demonstrated the increased neuronal expression of NLRP1 in the NeuN-positive neurons of mTLE patients (Figure 1c). After that, we further investigated the expression profiles of caspase-1. We found that the expression of caspase-1, an indicator of pyroptosis, showed a robust increase in hippocampus of chronic mTLE than that of controls (Figure 1d). Moreover, we have confirmed that the difference between the two control groups was not statistically significant (see Additional file 3: Figure S3).

### Inhibition of NLRP1-attenuated neuron pyroptosis in temporal lobe epilepsy rats

Next, we investigate the possible mechanism involved in the neuron pyroptosis with increased NLRP1 level. Toward this end, we knocked down brain NLRP1 expression of TLE rats by using *in vivo* nonviral RNA interference methodology [17]. To evaluate the silencing efficiency of siRNA infusion, the gene expression and protein level of NLRP1 protein were detected by quantitative real-time PCR and western blotting, respectively. Compared with control siRNA, NLRP1 siRNA significantly reduced the gene expression of NLRP1 in brain (see Additional file 4: Figure S4a). Consistent with the changes in gene expression, TLE rats infused with NLRP1 siRNA showed a dramatic reduction in hippocampus NLRP1 protein levels. Incidentally, the NLRP1 levels and gene expression between control-siRNA treated and No siRNA treated cells do not differ (see Additional file 4: Figure S4b), excluding an effect of siRNA transfection on gene expression or the protein level of NLRP1.

**Figure 2:**
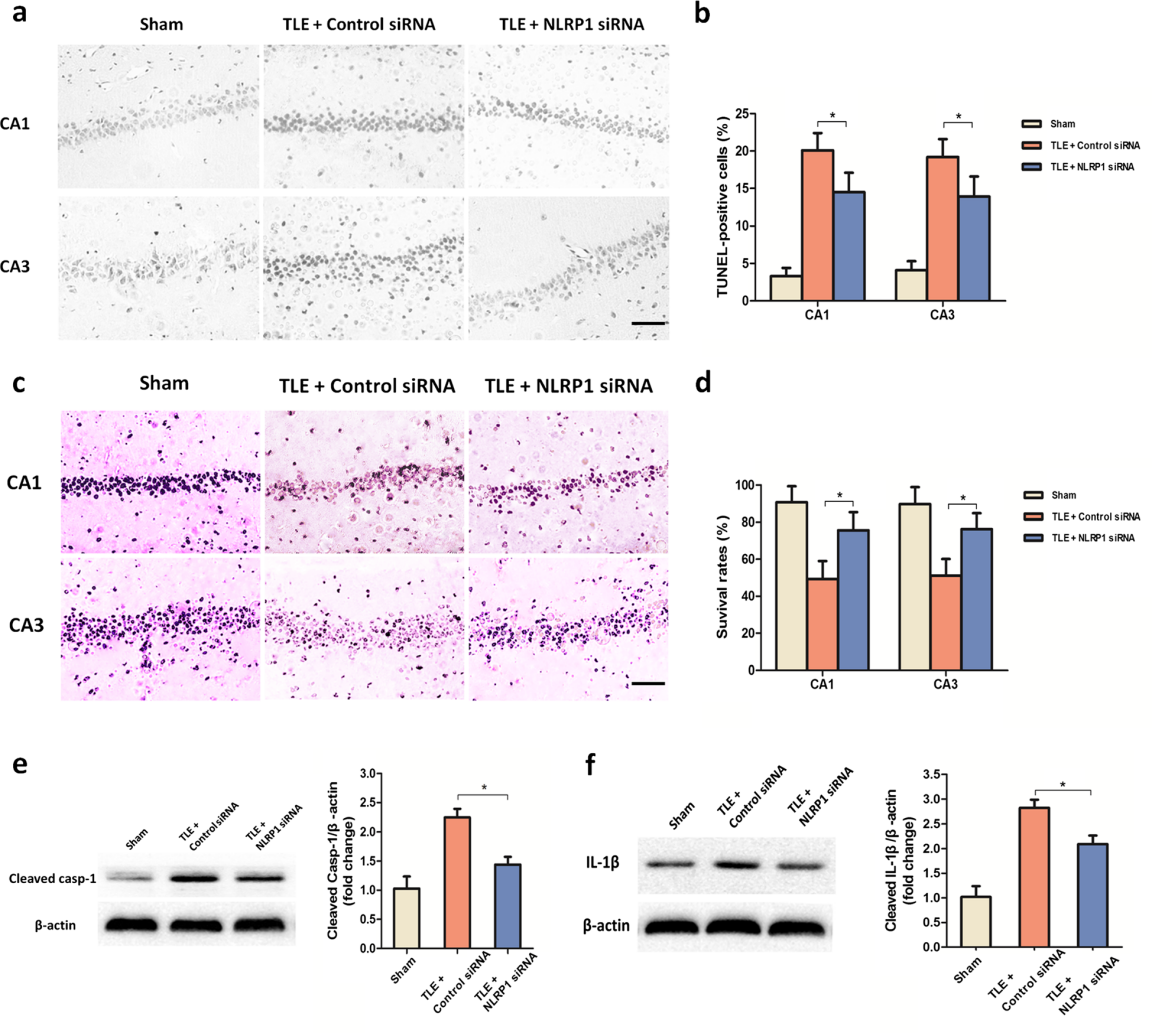
**NLRP1 inhibition attenuated neuronal pyroptosis in the temporal lobe epilepsy (TLE) rat brain. (a)** Neuronal pyroptosis was detected using the TUNEL staining assay in the CA1 region and CA3 region of rat hippocampus. Photos were converted to black and white to obtain a better contrast ratio. Neurons with deep black nuclei were identified as TUNEL-positive neurons. Scale bars: 20 μm. **(b)** Comparison of the percentage of TUNEL positive cells among the experimental groups. **(c)** Representative photo of Nissl-staining in CA1 region and CA3 region of rat hippocampus. Neurons with intact morphology were identified as surviving neurons. Scale bars: 20 μm. **(d)** Comparison of the percentage of survival neurons among the experimental groups. **(e)** Cerebral caspase-1 levels from rat hippocampus were detected by western blot analysis. β-actin was used as loading control. **(f)** The expression level of cleaved IL-1β (18KD) was detected by western blot analysis and quantified by densitometric measurement. β-actin was used as loading control. All data are shown as mean ± standard deviation. Obtained from six rats per group. *P <0.05, compared with the group of sham rat treated with control siRNA.

Then, we investigate the effects of NLRP1 inhibition on neuron pyroptosis in TLE rats. The TUNEL staining assay was firstly used to characterize the pyroptotic effects of NLRP1. Compared to the control-siRNA group, the increased number of TUNEL-positive cells in the hippocampus of TLE rats could be inhibited by NLRP1 siRNA treatment (Figure 2a, b). In addition, cresyl violet staining showed that NLRP1 siRNA led to less obvious neuronal loss in hippocampus of TLE rat brain (Figure 2c, d). We further examined the changes in caspase-1, which has been known to play a central role in the execution of pyroptosis. Western blot analysis was performed to measure the caspase-1 expression. As indicated in the Figure 2e, the NLRP1 siRNA treatment could reduce the active caspase-1 levels in TLE rats. In addition to pyroptosis, NLRP1 inflammasome is also responsible for caspase-1-dependent processing of the key pro-inflammatory cytokine IL-1β to an active secreted form [18,19]. In particular, the activation of IL-1β, which is involved in inflammatory cycle, is demonstrated to play a key role in TLE pathology [20]. Therefore, we also inspected the cerebral IL-1β levels by western blot. Similarly, NLRP1 siRNA treatment also changes the expression of active IL-1β (Figure 2f) in the brain of TLE rats.

### Inhibition of NLRP1 reduced seizure frequency and severity in temporal lobe epilepsy rats

To investigate the effects of NLRP1 siRNA on seizure frequency and severity in TLE rats, we assessed seizures of TLE rats during the first 24 h post-surgery and 6th week post-treatment. Our study demonstrated that sham rats after 6 weeks of treatment with NLRP1 siRNA or control siRNA showed no signs of seizure activity during the first 24 h post-surgery and/or 6th week post-treatment. In contrast, all rats (100%) in the TLE rats with control siRNA exhibited high scores of seizures within the first 24 h post-surgery and 77.8% of them had seizure activity on the 6th week post-treatment. Meanwhile, TLE rats with NLRP1 siRNA showed lower seizure activity as compared to the TLE group with control siRNA within the first 24 h (100%) and only 44.4% of them had seizure activity on the 6th week post-treatment and this difference versus TLE rats with control siRNA was significant for the 6th week post-treatment (Table 2a).

**Table 2:** **Numbers and rates of animals with seizures during the first 24 h postsurgery and on the 6th week post-treatment in experimental groups**

**(a) Results from sham and temporal lobe epilepsy rats after 6 weeks of treatment with NLRP1 siRNA or control siRNA**				
	**Within 24 h post-stimulation**		**On the 6th week post-treatment**	
	**Number**	**Rate (%)**	**Number**	**Rate (%)**
Sham + Control siRNA	0/18	0	0/18	0
Sham + NLRP1 siRNA	0/18	0	0/18	0
TLE + Control siRNA	18/18	100	14/18	77.8
TLE + NLRP1 siRNA	18/18	100	8/18	44.4^a^
**(b) Results from sham and TLE rats after 6 weeks of treatment with Caspase-1 siRNA or control siRNA**				
Sham + Control siRNA	0/18	0	0/18	0
Sham + Caspase-1 siRNA	0/18	0	0/18	0
TLE + Control siRNA	18/18	100	14/18	77.8
TLE + Caspase-1 siRNA	18/18	100^b^	7/18	38.9^b^

^a^ *P* <0.01 (versus TLE + Control siRNA).

^b^ *P* <0.01 (versus TLE + Control siRNA).

### Inhibition of Caspase-1 alleviated pyroptosis and seizure frequency and severity in temporal lobe epilepsy rats

**Figure 3:**
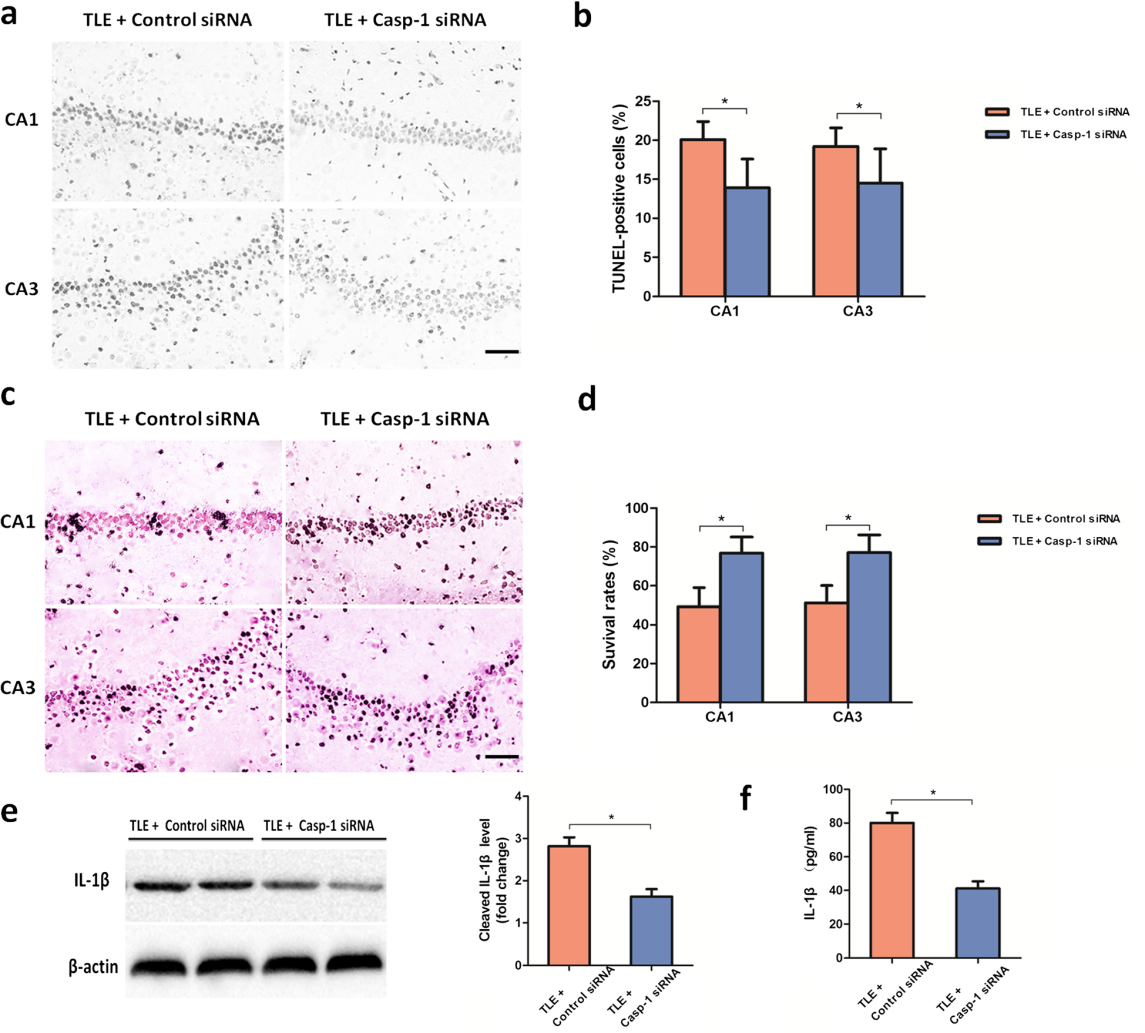
**Inhibition of Caspase-1 alleviated pyroptosis in temporal lobe epilepsy (TLE) rats.** Data from TLE rats after 6 weeks of treatment with caspase-1 siRNA or control siRNA using a mini-osmotic pump. **(a)** Neuronal pyroptosis was detected using the TUNEL staining assay in the CA1 and CA3 of rat hippocampus. Neurons with deep black nuclei were identified as TUNEL-positive neurons. Scale bars: 20 μm. **(b)** Quantitative analysis of the percentage of TUNEL positive cells among the experimental groups. **(c)** Representative photo of Nissl-staining in CA1 region and CA3 region of rat hippocampus. Neurons with intact morphology were identified as surviving neurons. Scale bars: 20 μm. **(d)** Comparison of the percentage of survival neurons among the experimental groups. *P <0.05 versus control siRNA treated group. **(e)** Western blot analysis of cleaved IL-1β in brain tissues and densitometrical quantification. β-actin was used as loading control. *P <0.05 versus control siRNA treated group. **(f)** The presence of IL-1β was measured by ELISA. All data are shown as mean ± standard deviation.

As caspase-1 play a central role in the process of neuron pyroptosis and IL-1β secretion, we also knocked down brain caspase-1 in TLE rats by *in vivo* nonviral RNA interference methodology for 6 weeks (see Additional file 1: Figure S1c,d). We found a marked reduction of TUNEL-positive cell densities in the CA1 and CA3 of TLE rat hippocampus (Figure 3a, b). Furthermore, cresyl violet staining showed that NLRP1 siRNA led to less obvious neuronal loss in hippocampus of TLE rat brain (Figure 3c, d). These results are consistent with the changes in NLRP1 siRNA-treated TLE rats, supporting that NLRP1 acts through caspase-1 to exert the observed effects. In addition, the expression of active IL-1β showed a decrease trend in the TLE rats with caspase-1 siRNA (Figure 3e, f).

As indicated from the Table 2b, sham rats after 6 weeks of treatment with caspase-1 siRNA or control siRNA showed no signs of seizure activity during the first 24 h post-surgery and/or 6th week post-treatment. While control siRNA-treated TLE rats showed high seizure frequency and severity. In contrast, caspase-1 siRNA treatment could largely reduce seizure frequency and severity in TLE rats. Only 38.9% of them had seizure activity on the 6th week post-treatment and this difference versus TLE rats with control siRNA was significant.

## Discussion

Temporal lobe epilepsy (TLE) is often characterized pathologically by severe neuronal loss in the hippocampus. Understanding the mechanisms of cell death is a key for preventing the neurodegeneration associated with TLE. However, the involvement of neuronal death to the epileptogenic process has yet to be fully determined. Recent studies shown that the activation of NLRP1 inflammasome can generate a functional caspase-1-containing inflammasome *in vivo* to drive the proinflammatory programmed cell death termed ‘pyroptotic death’ [5], which have key roles in the pathogenesis of neurological disorders [4,21,22]. NLRP1 is also highly expressed in pyramidal neurons of the brain [8]. In addition, inflammasome activation especially the NLRP1 is under current investigation across a broad spectrum of neurological diseases, including infections, acute sterile brain injury and chronic neurodegenerative diseases [23]. However, to the best of our knowledge, there are no reported studies that performed a detailed identification and validation of NLRP1 inflammasome during the epileptogenic process. Hence, we hypothesized that the activation of NLRP1-inflammasome may have key roles in the pathogenesis of mTLE, in which neuronal death combined with inflammation contributes powerful pathogenetic forces [24].

Our study was for first time to examine whether the NLRP1-dependent pyroptosis is a potential mechanism in TLE pathogenesis in refractory medial temporal lobe epilepsy patients. We compared expression of NLRP1 and caspase-1 in resected hippocampus from patients with intractable mTLE to matched control samples. The present study detected upregulated NLRP1 and caspase-1 levels within mTLE samples than controls. Meanwhile, the cellular localization of NLRP1 on the neuron in the mTLE patient brains was demonstrated by the double immunofluorescence staining, consistent with the findings from the other studies [9]. These elevated NLRP1 inflammasome levels may be induced by potassium (K^+^) efflux, which is causative of the generation or spread of seizure activity in TLE [25,26].

Moreover, we knocked down brain NLRP1 expression by implanting mini-osmotic pumps for direct infusion of siRNA to investigate its role on neuronal pyroptosis in the TLE rat model evoked by amygdala stimulation. This amygdala stimulation model of chronic TLE in rats provides a useful tool for studies aimed at understanding the mechanisms of TLE and exploring the new therapeutic strategy for this disease [27,28]. RNA interference technology has emerged as a potentially superior alternative to the traditional approaches for assessing gene function in adult animals to understand the genes implicated in neuropsychiatric disorders [17,29]. Moreover, application of the nonviral infusion of siRNA into the ventricular system could achieve a widespread sequence-specific gene knockdown in the brain [30]. For the characteristics of rapid, inexpensive, and specific knockdown of target genes in the whole brain, this method could provide a useful tool to accelerate the functional investigation of broadly expressed target genes implicated in neurological disorders. In our study, we found that this approach effectively downregulates the levels of NLRP1 mRNA and protein in TLE rat brain. Meanwhile, compared to no siRNA treated TLE rat, the treatment with control siRNA did not alter cerebral NLRP1 mRNA and protein levels, thus excluding an effect of pump-mediated infusion on NLRP1 expression levels. For the first time, we revealed that NLRP1 siRNA treatment could significantly reduce TUNEL-positive cell densities and the active caspase-1 expression levels in TLE rats, supporting a fundamental role for NLRP1-mediated pyroptosis in TLE-induced neuronal losses in the hippocampus.

Meanwhile, we should notice that NLRP1 inflammasome is also responsible for caspase-1-dependent processing of the key pro-inflammatory cytokine IL-1β to an active secreted form [18,19]. Pro-inflammatory cytokines, including IL-1β, are known to modulate effects of neurotoxic neurotransmitters discharged during excitation or inflammation in the central nervous system (CNS) [31]. Moreover, current studies pointed out that the inflammatory mechanism contributes powerful pathogenetic forces in the process of TLE [10]. And recent studies found that IL-1β was proconvulsant in animal models of TLE, increasing the time spent in seizures and reducing the onset time of the first seizure [32]. Hence, the elevated levels of IL-1β in TLE are believed to serve as a part of inflammatory cycle that regulates TLE pathology. Our current research has found that NLRP1 siRNA treatment could change the expression of active IL-1β in the brain of TLE rat. However, we should notice that there may be another pathway involved in caspase-1-dependent processing of the key pro-inflammatory cytokine IL-1β. Previous studies have pointed out that NLRP3 inflammasome also can activate caspase-1 to induce IL-1β secretion [28]. In contrast to the activation of NLRP3, inflammasome mainly expressed in microglia, which is essential for the secretion of pro-inflammatory cytokines and subsequent inflammatory events, the NLRP1 mainly expressed in neuron, which is essential for the pyroptotic cell death in TLE pathogenesis.

To further investigate whether the NLRP1 acts through caspase-1 to exert the observed effects of pyroptosis, we also knocked down brain caspase-1 by this *in vivo* nonviral RNA interference methodology in TLE rats. Consistent biochemical results were found between caspase-1 siRNA and NLRP1 siRNA-treated TLE rats, supporting that NLRP1 acts through caspase-1 signaling to exert the effects of pyroptosis. The only difference was that silencing caspase-1 could markedly reduce active IL-1β expression in the TLE rats. As we all know, caspase-1 is a critical pathway by which inflammasomes contribute to the downstream effects. Beside the NLRP1, other inflammasome such as NLRP3 also can activate caspase-1 to induce IL-1β secretion.

This study also has some limitations. For example, we cannot rule out the possibility that the 6-week infusion of NLRP1 siRNA is not enough to cause obvious changes in TLE-like neuropathology. However, the limited duration (maximum 6 weeks) of osmotic pumps suitable for rat prevented us from observing long-term effects of NLRP1 knockdown. Second, there are limited ways to stimulate or stably overexpress NLRP1 in brain. Hence, we cannot provide more direct evidence on the role of NLRP1 in neuroinflammation and pyroptotic cell death here. However, it is still worth mentioning that our findings have important clinical implications. Although current available interventions can provide benefits for TLE, none prevents or cures the disease. Thus, the inhibition of neuronal pyroptosis is a neuroprotective response and pyroptosis could be restrained to open exciting new therapeutic perspectives for TLE. Thus, because inhibition of NLRP1 inflammasome had several potentially beneficial effects on neuronal pyroptosis and inflammation response in the TLE process, modulation of NLRP1 inflammasome may be explored as a promising strategy for the development of TLE therapy.

## Conclusions

**Figure 4:**
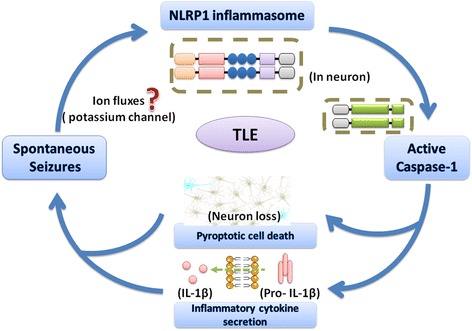
**NLRP1 inflammasome contributes to pyroptosis in chronic temporal lobe epilepsy.** High NLRP1 levels were found in pyramidal neurons of the brain. The spontaneous seizures may set fire to neuronal NLRP1 inflammasome via potassium efflux and other channels. Then, the activation of NLRP1 inflammasome leads to the caspase-1-mediated pyroptosis and secretion of IL-1β, which ultimately induces TLE pathology through several downstream effects in brain. Our current study mainly indicated that the caspase-1-induced neuronal pyroptosis provides a molecular basis for the spontaneous seizures in TLE process.

Our present study first demonstrates that cerebral expression of NLRP1 was upregulated in refractory medial temporal lobe epilepsy patients. The increase in NLRP1 levels can activate caspase-1 signaling responsible for neuronal pyroptosis and inflammation cytokine release (Figure 4). Using the pump-mediated *in vivo* infusion of nonviral siRNA to knock down NLRP1 in the brain of TLE rats, our study further indicated that inhibition of NLRP1 inflammasome represents a potential clinical benefit of therapeutic interventions that target inflammasome assembly and activity.

## Additional files

Additional file 1: Figure S1. Magnetic resonance imaging (MRI) manifestations of all cases in the temporal lobe epilepsy (TLE) patient group. (a) Case 1 showed right hippocampal sclerosis. (b) Case 2 showed left hippocampal sclerosis. (c) Case 5 showed right hippocampal sclerosis. (d) Case 6 showed left hippocampal degenerative atrophy. Additional file 2: Figure S2. Positron emission tomography (PET) manifestations of cases in the temporal lobe epilepsy (TLE) patient group. (a) Case 1 showed low metabolism in the right temporal lobe. (b) Case 2 showed low metabolism in the left temporal lobe. (c) Case 5 showed low metabolism in the right temporal lobe. (d) Case 6 showed low metabolism in the left temporal lobe. Case 3 and Case 4 did not receive PET test. Additional file 3: Figure S3. Expression of NLRP1 and caspase-1 in the neurons of two control groups. (a) Cerebral NLRP1 and NeuN levels from two controls were detected by western blot analysis. β -actin was used as loading control. Levels of NLRP1, NeuN and NLRP1/NeuN were quantified by densitometric measurement. Values are the mean ± standard deviation. (b) The expression level of active caspase-1 (20KD) was analyzed using the western blot assay. n = 6 individuals per group. Control 1 indicates the normal temporal cortex tissues; Control 2 indicates postmortem control hippocampal tissue. Additional file 4: Figure S4. Short interfering RNA (siRNA) targeting NLRP1 or caspase-1 effectively downregulated NLRP1 or caspase-1 in brain of TLE rat model. (a) Messenger RNA levels of NLRP1 in brain of temporal lobe epilepsy (TLE) rats after a 6-week infusion of artificial cerebrospinal fluid (aCSF), control siRNA or NLRP1 siRNA. (b) Protein levels of NLRP1 in the brain of the TLE rats after a 6-week infusion of artificial cerebrospinal fluid (aCSF), control siRNA, or NLRP1 siRNA. Data are expressed as a fold change relative to sham group. (c) Messenger RNA levels of caspase-1 in brain of TLE rats in sham, 6-week infusion of control siRNA, or caspase-1 siRNA groups. (d) Protein levels of caspase-1 in brain of TLE rats in sham, 6-week infusion of control siRNA or caspase-1 siRNA groups. Data are expressed as a fold change relative to TLE rats infused with control siRNA. Columns represent mean ± standard deviation. n = 6 rats per group.

## Abbreviations

ELISA: enzyme-linked immunosorbent assay
HS: hippocampal sclerosis
IL: interleukin
NLRP1: nucleotide binding and domain-like receptor family pyrin domain-containing 1
siRNA: small interfering RNA
PCR: polymerase chain reaction
TLE: temporal lobe epilepsy
TUNEL: terminal deoxynucleotidyl transferase-mediated dUTP end-labeling
