# Supplementary figures and images for: NLRP1 inflammasome is activated in patients with medial temporal lobe epilepsy and contributes to neuronal pyroptosis in amygdala kindling-induced rat model
Source: J Neuroinflammation. 2015 Jan 28;12:18. doi: 10.1186/s12974-014-0233-0 (PMC4314732; doi:10.1186/s12974-014-0233-0)

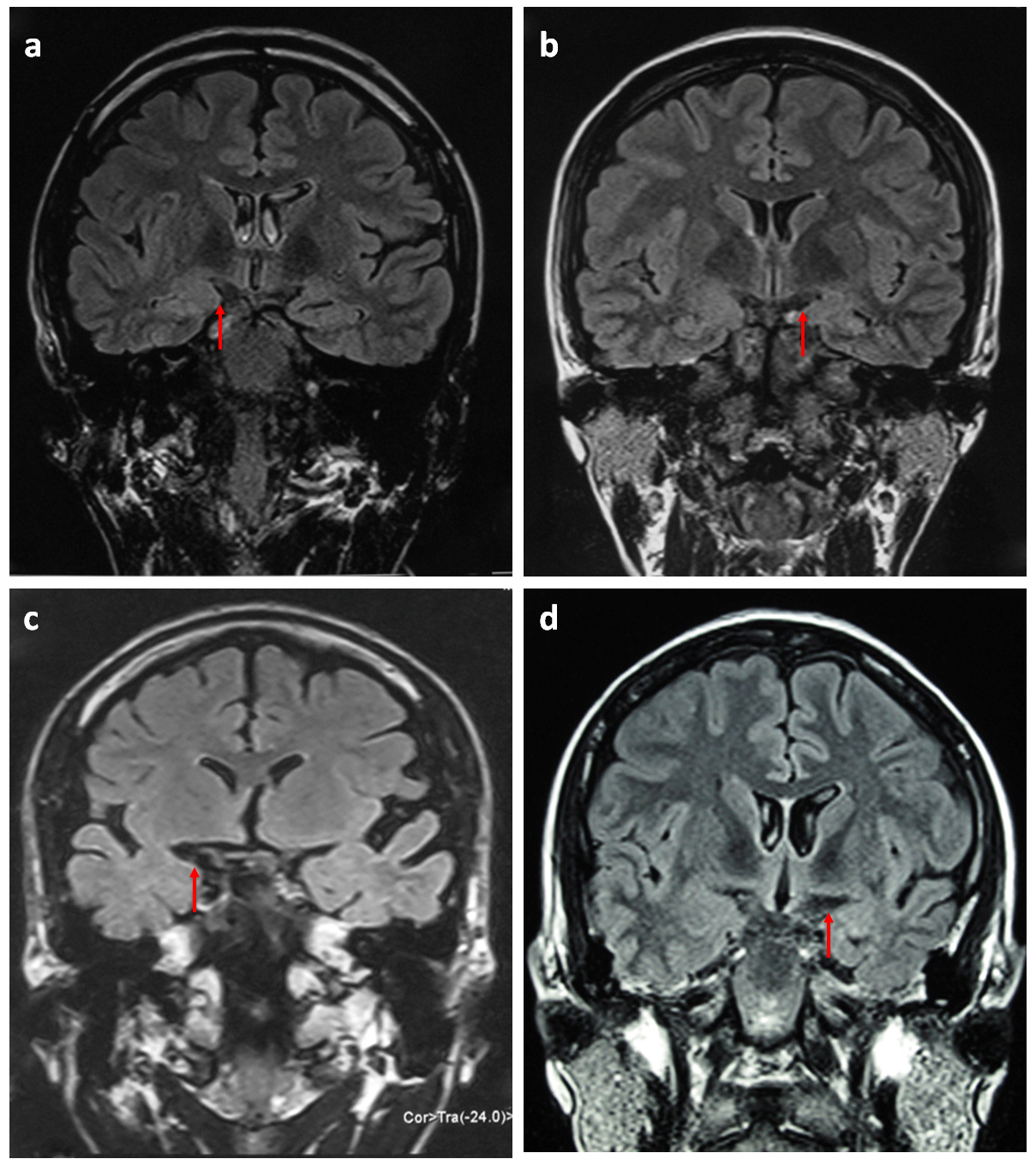

Supplement: Additional file 1: Figure S1. — Magnetic resonance imaging (MRI) manifestations of all cases in the temporal lobe epilepsy (TLE) patient group. (a) Case 1 showed right hippocampal sclerosis. (b) Case 2 showed left hippocampal sclerosis. (c) Case 5 showed right hippocampal sclerosis. (d) Case 6 showed left hippocampal degenerative atrophy. [file 12974_2014_233_MOESM1_ESM.tiff]

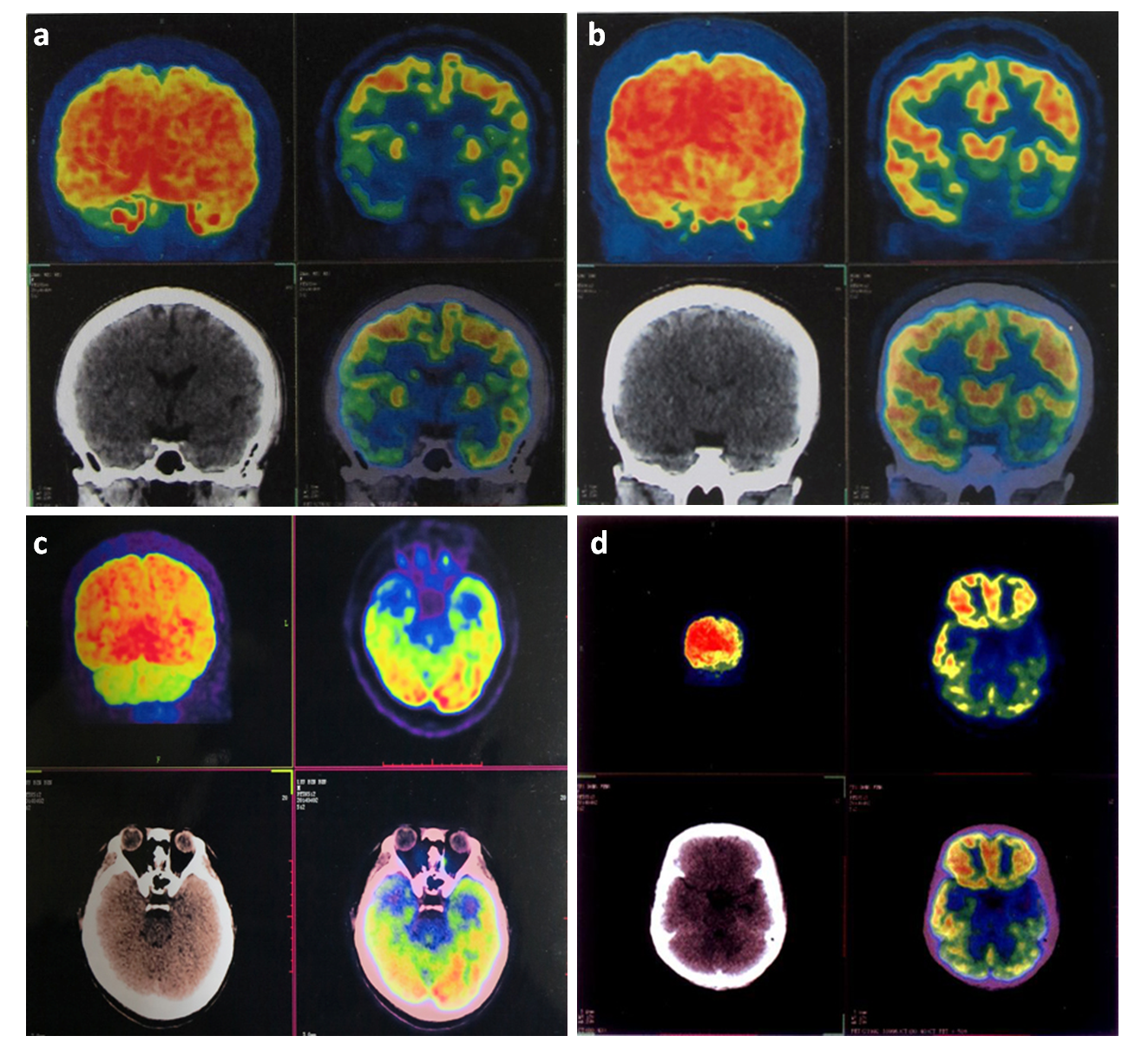

Supplement: Additional file 2: Figure S2. — Positron emission tomography (PET) manifestations of cases in the temporal lobe epilepsy (TLE) patient group. (a) Case 1 showed low metabolism in the right temporal lobe. (b) Case 2 showed low metabolism in the left temporal lobe. (c) Case 5 showed low metabolism in the right temporal lobe. (d) Case 6 showed low metabolism in the left temporal lobe. Case 3 and Case 4 did not receive PET test. [file 12974_2014_233_MOESM2_ESM.tiff]

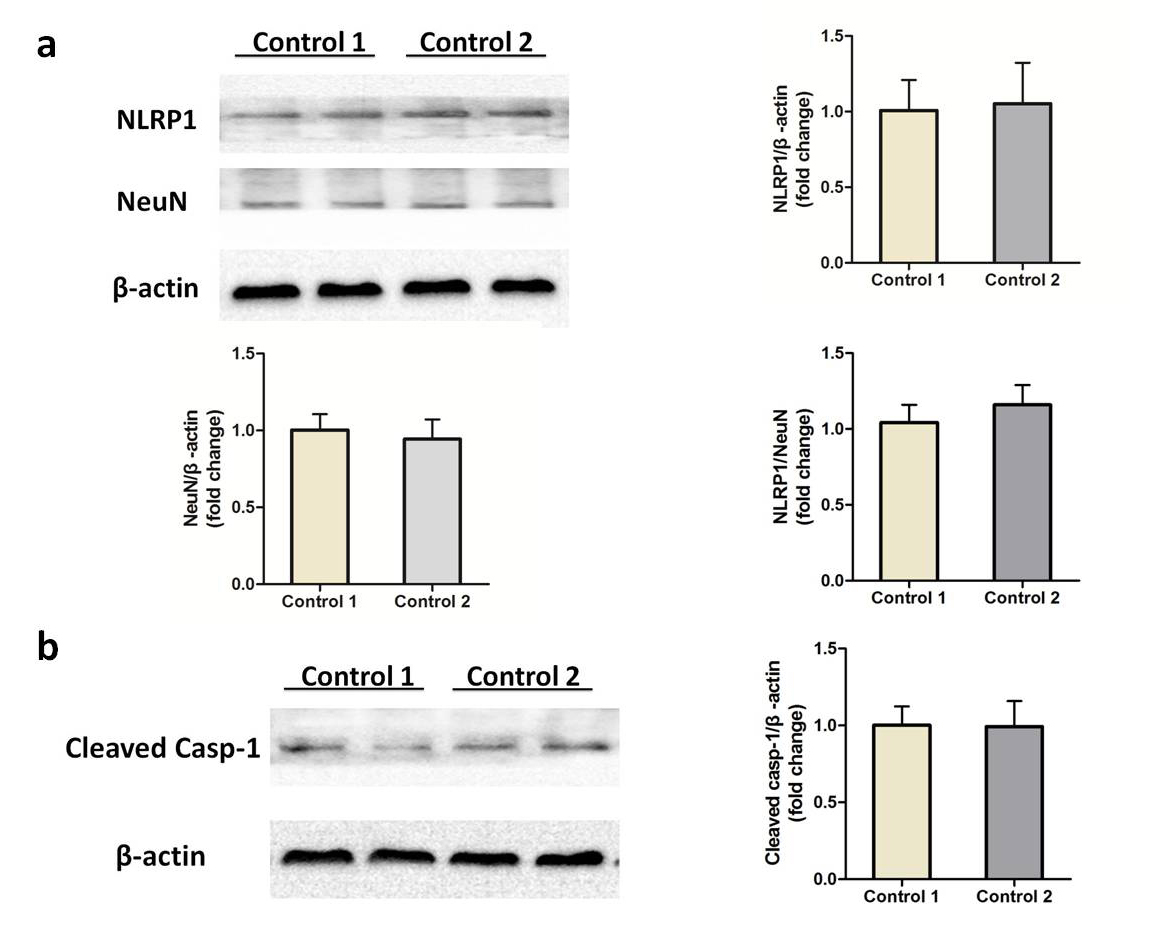

Supplement: Additional file 3: Figure S3. — Expression of NLRP1 and caspase-1 in the neurons of two control groups. (a) Cerebral NLRP1 and NeuN levels from two controls were detected by western blot analysis. β -actin was used as loading control. Levels of NLRP1, NeuN and NLRP1/NeuN were quantified by densitometric measurement. Values are the mean ± standard deviation. (b) The expression level of active caspase-1 (20KD) was analyzed using the western blot assay. n = 6 individuals per group. Control 1 indicates the normal temporal cortex tissues; Control 2 indicates postmortem control hippocampal tissue. [file 12974_2014_233_MOESM3_ESM.tiff]

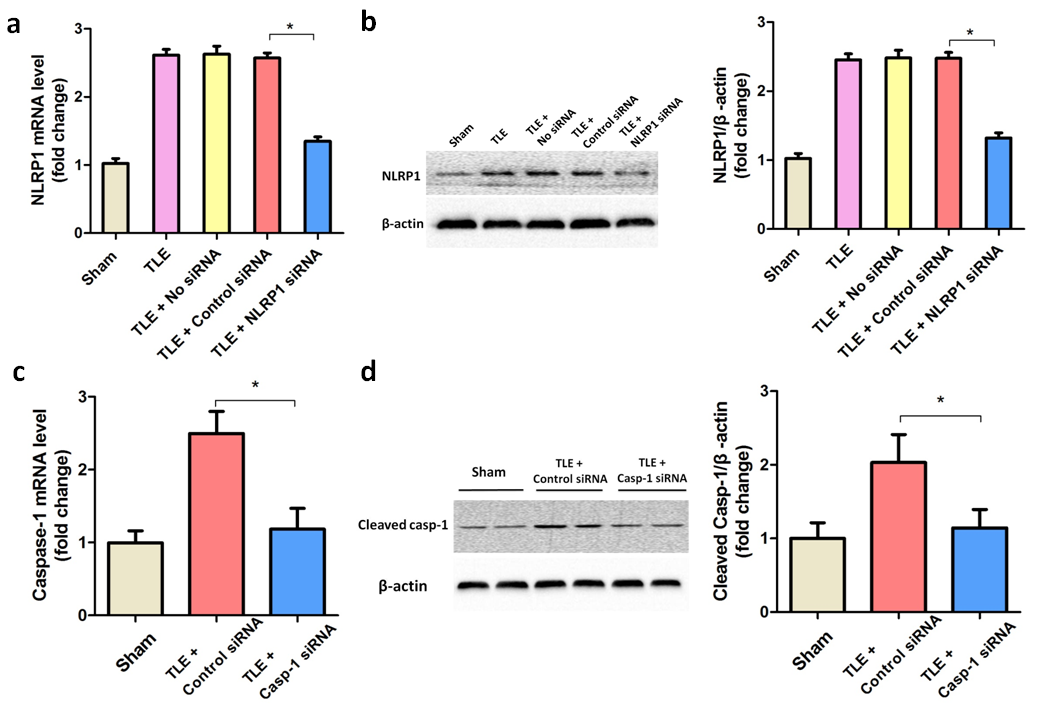

Supplement: Additional file 4: Figure S4. — Short interfering RNA (siRNA) targeting NLRP1 or caspase-1 effectively downregulated NLRP1 or caspase-1 in brain of TLE rat model. (a) Messenger RNA levels of NLRP1 in brain of temporal lobe epilepsy (TLE) rats after a 6-week infusion of artificial cerebrospinal fluid (aCSF), control siRNA or NLRP1 siRNA. (b) Protein levels of NLRP1 in the brain of the TLE rats after a 6-week infusion of artificial cerebrospinal fluid (aCSF), control siRNA, or NLRP1 siRNA. Data are expressed as a fold change relative to sham group. (c) Messenger RNA levels of caspase-1 in brain of TLE rats in sham, 6-week infusion of control siRNA, or caspase-1 siRNA groups. (d) Protein levels of caspase-1 in brain of TLE rats in sham, 6-week infusion of control siRNA or caspase-1 siRNA groups. Data are expressed as a fold change relative to TLE rats infused with control siRNA. Columns represent mean ± standard deviation. n = 6 rats per group. [file 12974_2014_233_MOESM4_ESM.tiff]
